# Electron carriers increase electricity production in methane microbial fuel cells that reverse methanogenesis

**DOI:** 10.1186/s13068-018-1208-7

**Published:** 2018-07-25

**Authors:** Ryota Yamasaki, Toshinari Maeda, Thomas K. Wood

**Affiliations:** 1 Department of Chemical Engineering, Pennsylvania State University, University Park, PA 16802-4400 USA; 2 Department of Biological Functions Engineering, Kyushu Institute of Technology, 2-4 Hibikino, Wakamatsu, Kitakyushu, 808-0196 Japan

**Keywords:** Microbial fuel cells, Anaerobic, *Geobacter*, Methane, Power density

## Abstract

**Background:**

We previously reversed methanogenesis in microbial fuel cells (MFCs) to produce electricity for the first time from methane by combining an engineered archaeal strain that produces methyl-coenzyme M reductase from unculturable anaerobic methanotrophs (to capture methane and secrete acetate) with *Geobacter sulfurreducens* (to produce electrons from the generated acetate) and methane-acclimated sludge (to provide electron shuttles).

**Results:**

Here, the power density in MFCs was increased 77-fold to 5216 mW/m^2^ and the current density in MFCs was increased 73-fold to 7.3 A/m^2^ by reducing the surface area of the cathode (to make reasonable comparisons to other MFCs), by changing the order the strains of the consortium were added to the anode compartment, and by adding additional electron carriers (e.g., humic acids and cytochrome C).

**Conclusions:**

This power density and current density are comparable to the best for any MFC, including those with *Shewanella* and *Geobacter* spp. that utilize non-gaseous substrates. In addition, we demonstrate the methane MFC may be used to power a fan by storing the energy in a capacitor. Hence, MFCs that convert methane to electricity are limited by electron carriers.

**Electronic supplementary material:**

The online version of this article (10.1186/s13068-018-1208-7) contains supplementary material, which is available to authorized users.

## Background

The microbial fuel cell (MFC) is a device that utilizes microorganisms to convert the chemical energy of organic matter into electric energy [1]. An MFC consists of an anode, where electrons generated from the oxidation of the fuel (organic matter) by microorganisms are collected, and the cathode, where electrons move to be consumed by the reduction reaction of an oxidizing agent; the electricity generated may be stored in capacitors [2].

Various microorganisms produce electricity such as *Geobacter*, *Shewanella*, and *Rhodoferax* spp. from various organics; for example, *G. sulfurreducens* generates electricity from hydrogen and acetate [3], whereas *S. putrefaciens* generates electricity from lactate and pyruvate [4] and *R. ferrireducens* generates electricity from glucose [5]. In these bacteria, electrons may be exported through a pilus (nanowires) [6], through cell membranes as multiple-heme complexes [7], or by molecular carriers generated by microorganisms [7].

The potential difference between the anode and the cathode is the driving force for MFC power generation: the higher the potential energy of the anode, the larger the supplied electric energy from Ohm’s law (V = IR). MFCs are highly efficient at (i) producing electricity [8], using a wide variety of substrates such as acetate [3] and glucose [9], and at (ii) treating wastewater [10], because MFCs produce primarily electricity instead of heat [7]. However, MFCs do not produce sufficient power for many applications [7] and typically, a power density of around 1240–2800 mW/m^2^ is obtained [9], although power densities as high as 7200 mW/m^2^ may be obtained (current density of 15 A/m^2^) [11]. Hence, it is important to increase power density to allow MFCs to have broader applications [7].

We demonstrated recently [8] that a methane MFC can be made by utilizing an engineered consortium that combines (i) an engineered archaeal strain, *Methanosarcina acetivorans* AA/pES1MAT *mcr3*, to capture methane and convert it to acetate with (ii) *Geobacter sulfurreducens* PCA, to convert acetate to electrons, and (iii) anaerobic sludge, to provide electron shuttles. *M. acetivorans* AA/pES1MAT *mcr3* produces methyl-coenzyme M reductase from unculturable anaerobic methanotrophs [12]; in effect, methanogenesis was reversed in methanogen *M. acetivorans* to allow us to capture methane [12]. *M. acetivorans* AA/pES1MAT *mcr3* has also been used by us to produce lactate efficiently from methane [13]. It was necessary to utilize an engineered methanogen for our MFC because the anaerobic methanotrophs that capture 300–400 million tons of methane per year in sediments [14] have not been cultured successfully [15]. We activated the sludge to select the sludge components that are active in methane. Critically, we showed the activated sludge in our MFC could be replaced by *Paracoccus denitrificans,* which has been shown to provide electron carriers and can be replaced by the electron-carrier humic acids (with a 45% reduction in current) [8], which suggests that the electron carrier may be rate limiting in our system.

Cytochrome C is utilized to transfer electrons in many microorganisms, animals, and plants [16, 17]. *G. sulfurreducens* also has cytochrome C to transfer electrons to external Fe^3+^ [18]. Therefore, adding cytochrome C as an external electron mediator to MFCs may enhance external electron transport in the anode chamber.

In this paper, our goal was to determine what is rate limiting in the methane MFC to enhance its power production. To discern this, we varied consortial members (e.g., *Geobacter* spp.), changed media components (e.g., high concentration of salts, adding acetate), added electron carriers (e.g., humic acids, cytochrome C), changed the cathode size, changed the anode material, and changed the order of adding members of the consortium to the reactor. We determined that increasing the electron-carrier humic acids significantly increases the current density and power density such that they are comparable to the highest levels achieved in a MFC.

## Results

### MFC system

**Fig. 1:**
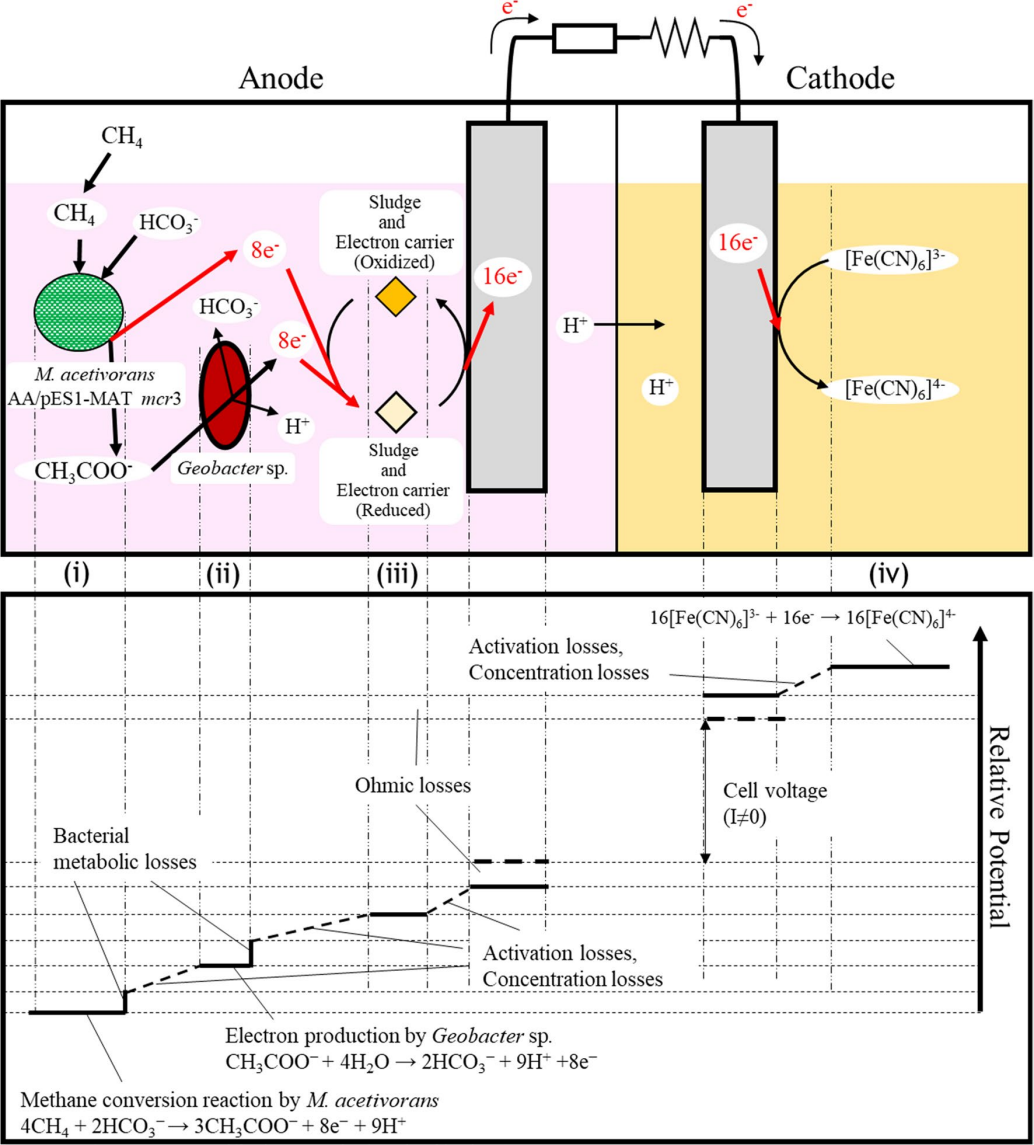
MFC schematic and voltage diagram. The upper panel illustrates current flow in the MFC, from the oxidation of methane in the anode via *M. acetivorans* AA/pES1MAT *mcr3* (“*M. acetivorans*”), *Geobacter* sp., sludge, and electron carriers such as humic acids, to electron consumption in the cathode. In the anode, (i) methane is converted to acetate, which is secreted, by *M. acetivorans* AA/pES1MAT *mcr3*. Next, (ii) electrons are produced from acetate by *Geobacter* sp. (iii). These electrons are transferred to the carbon brush electrode by electron carriers. Electrons are moved to the cathode through a voltmeter and external resistance (1000 Ω) and (iv) consumed by [Fe(CN)_6_]^3−^. The lower panel illustrates the voltage (potential energy) for each reaction (adapted from Nagatsu et al. [36]) The first potential band (left end) is for the conversion of methane and electron production reaction by *M. acetivorans* AA/pES1MAT *mcr3*. The second potential band is for the electron production reaction from acetate by *Geobacter* sp. These two reactions have bacterial metabolic losses. The third potential band is for the step provided by the sludge and its electron carriers. The last potential band (right end) is for electron consumption by [Fe(CN)_6_]^3−^. There is an activation or concentration loss in proceeding through the each reaction. The *y*-axis of this panel is arbitrary

To obtain higher electricity levels from the MFC, it is necessary to either produce higher voltages as indicated by Ohm’s law (V = IR) or reduce the resistance. The base case #1 for this MFC work consisted of adding *M. acetivorans* AA/pES1MAT *mcr3* and *G. sulfurreducens* first to the reactor, and once the voltage was reduced to 150 mV, adding activated sludge. A schematic of the MFC showing the electron flow for the engineered consortium is shown in Fig. 1. The base case #1 replicates produced an average voltage of 670 mV (Table 1). We then varied the medium, consortia, electrode, and provided electron carriers to increase the system voltage.

**Table 1:** Maximum voltage (mV) in the MFC reactors

MFC reactor	Maximum voltage (mV)
Base case #1	670 ± 60
Trace element solution (1× → total 5×) with *G. metallireducens*	272
Vitamin solution (1× → total 5×) with *G. metallireducens*	275
Cysteine–HCl (3.2 mM → total 16 mM) with *G. metallireducens*	524
Na_2_S (1 mM → total 5 mM) with *G. metallireducens*	649
Base case #1 without cysteine–HCl	757
Base case #1 + sodium acetate (10 mM)	562
Base case #1 + *D. vulgaris*	622
Base case #1 + Baar’s medium	623
*G. metallireducens*	641.0 ± 0.7
Base case #1 + *G. metallireducens* (mix of *G. sulfurreducens* and *G. metallireducens*)	158
Base case #1 + cytochrome C from equine heart (20 µM)	500 ± 200
Base case #1 + cytochrome C from *S. cerevisiae* (20 µM)	690 ± 70
Base case #1 + humic acids (0.5%)	660 ± 60
*G. sulfurreducens* at start then sludge + *M. acetivorans* (base case #2)	721.5 ± 0.7
Base case #1 with a Pt wire anode	663 ± 7

Base case #1 is *M. acetivorans* AA/pES1MAT *mcr3* (“*M. acetivorans*”) and *G. sulfurreducens* added first followed by sludge once the voltage was below 150 mV. *G. metallireducens* replaced *G. sulfurreducens* upon inoculation. Sodium acetate (10 mM) was added to the base case #1 with sludge. *D. vulgaris*, Baar’s medium, and *G. metallireducens*, and were added to the base case #1 after it reached the maximum voltage. Cytochrome C from equine heart (20 µM), cytochrome C from *S. cerevisiae* (20 µM), and humic acids (0.5%) were added to the base case #1 with sludge. Base case #1 was altered by adding *G. sulfurreducens* at the start then adding sludge + *M. acetivorans* once the voltage was reduced to 150 mV. The anode electrode was replaced by Pt wire (surface area 1 × 10^−5^ m^2^). The cathode electrode diameter was 38 mm (surface area is 0.00227 m^2^). The external resistance was 1000 Ohm

### Medium variation and pH

To determine if any medium component was limiting electricity production, we varied the composition of the MFC medium, HSNR (Additional file 1: Table S1), which includes trace elements, vitamins, cysteine–HCl (3.2 mM), and Na_2_S (1 mM). After increasing by 5× the trace element solution, the vitamin solution, the cysteine–HCl, and Na_2_S, the maximum voltages were 272, 275, 524 and 649 mV, respectively (Table 1). Also, when cysteine–HCl was removed, a voltage of 757 mV was obtained (Table 1). Because *M. acetivorans* AA/pES1MAT *mcr3* converts methane to acetate which is utilized by *G. sulfurreducens* [8], we also tried adding sodium acetate (10 mM) to the base reactor; however, the voltage was reduced (562 mV, Table 1). Therefore, we concluded that the medium composition was not affecting the voltage substantially. Additionally, the pH of the anode chamber of the MFC reactor was measured for the MFC medium (before inoculation) and for two reactors (after 3 and 6 months) for the base case #2 reactor set with 3.3% humic acids (Table 2) and found to not vary (pH approximately constant at 7.4).

**Table 2:** MFC voltage (mV), current density (A/m^2^) and power density (mW/m^2^) after adding humic acids as additional electron carriers with a MFC with a small cathode (surface area is 50 × 10^−6^ m^2^)

MFC reactor	Voltage (mV)	Current density (A/m^2^)	Fold change	Power density (mW/m^2^)	Fold change
Base case #1	670 ± 60	0.10 ± 0.01	1	68 ± 6	1
Base case #2	721.5 ± 0.7	0.6 ± 0.1	6	450 ± 90	7
Base case #2 + humic acids (total 0.5%)	712 ± 4	2.1 ± 0.4	21	1400 ± 200	21
Base case #2 + humic acids (total 3.3%)	750 ± 60	6 ± 1	60	4700 ± 800	69
Base case #2 + cytochrome C from *S. cerevisiae* (20 µM)	732 ± 2	0.97 ± 0.07	10	710 ± 40	10

The base case #1 is *M. acetivorans* AA/pES1MAT *mcr3* (“*M. acetivorans”*) and *G. sulfurreducens* was added first followed by sludge once the voltage was below 150 mV and using 38-mm-diameter cathode electrode (surface area is 0.00227 m^2^). The base case #2 consists of *G. sulfurreducens* added at the start followed by the addition of sludge + *M. acetivorans* AA/pES1MAT *mcr3* once the voltage was reduced below 150 mV and using a small cathode electrode (surface area is 50 × 10^−6^ m^2^). The additional electron carriers (humic acids) were added after obtaining the first maximum voltage; 0.5% humic acids were added after 7–8 days and 3.3% humic acids were added after 13–14 days of adding sludge and *M. acetivorans*. Cytochrome C from *S. cerevisiae* (20 µM) was added as an additional electron carrier after obtaining the first maximum in voltage after adding sludge and *M. acetivorans* AA/pES1MAT *mcr3*. The external resistance was 1000 Ohm

### Consortia variation and electrode

Because sulfate-reducing bacteria (SRB) are found in conjunction with methanogens in natural environments [19], we tried adding the representative SRB *Desulfovibrio vulgaris*. This culture was added (2 mL, OD_600_ = 0.5) into the base case #1 reactor after it reached the maximum voltage. Baar’s medium (without *D. vulgaris*) was also added as a negative control. The maximum voltage of adding *D. vulgaris* culture was 622 mV and that of adding Baar’s medium was 623 mV; hence, there were no substantial changes in voltage upon adding SRB.

Because the *Geobacter metallireducens* pili have 5000-fold higher conductivity than the *G. sulfuriducens* pili [20], we replaced *G. sulfurreducens* with this strain but did not obtain substantial increases in the average voltage (641 mV, Table 1). Because co-metabolism can be established by joint electron transfer between *G. sulfurreducens* and *G. metallireducens*, we also tried both *Geobacter* strains simultaneously in the MFC; however, the voltage was reduced considerably (158 mV, Table 1). We also varied the order of strain addition to the MFC by adding *G. sulfurreducens* at the start, to allow it to form a biofilm on the anode, then adding sludge and *M. acetivorans* once the voltage was reduced to 150 mV. In this case, the voltage improved to 722 mV (Table 1).

To investigate the importance of the anode, the anode electrode of base case #1 reactor was replaced with a platinum wire electrode (surface area 1 × 10^−5^ m^2^). However, the voltage was unchanged and similar to that of base case #1 (663 mV, Table 1).

### Addition of electron carriers

To explore whether our MFC was limited by electron carriers, we tried adding (i) two types of cytochrome C (from equine heart and from *Saccharomyces cerevisiae*) and (ii) two concentrations of humic acids (0.5 and 3.3%). The addition of cytochrome C from equine heart at 20 µM with sludge did not improve the average voltage (500 mV, Table 1). However, cytochrome C from *S. cerevisiae* improved the average voltage (690 mV, Table 1). Also, when humic acids were added (0.5%) to the base case #1 reactor with sludge, the voltage was not improved substantially (660 mV, Table 1).

### Current density and power density in the MFC

Because the highest voltage was obtained in the MFC upon adding the electron carrier cytochrome C (from *S. cerevisiae*), we explored whether current density was affected by the addition of cytochrome C (from *S. cerevisiae*) or humic acids, i.e., we explored whether current was increased as a result of reduced resistance. First, the base case #1 current density and power density of the MFC was determined to be 0.1 A/m^2^ and 68 mW/m^2^ (Table 2).

To increase the current density and to be able to compare our results to that of other MFCs using substrates other than methane, a 45-fold smaller cathode electrode was employed (carbon cloth, 5 mm × 5 mm, surface area of 5 × 10^−5^ m^2^ compared to the 227 × 10^−5^ m^2^ of base case #1). In this MFC reactor, *G. sulfurreducens* was added initially, then sludge and *M. acetivorans* were added once the system voltage was less than 150 mV because this order of strain addition showed one of the highest voltages (722 mV, Table 1). This reactor was defined as “base case #2” (i.e., *G. sulfurreducens* added first and the small electrode). The current density and power density of the base case #2 system was 0.6 ± 0.1 A/m^2^ and 450 ± 90 mW/m^2^, a sixfold improvement over base case #1 over the large electrode (Table 2).

When the electron carrier cytochrome C (from *S. cerevisiae*) was added to the base case #2 reactor at 20 µM, the current density increased tenfold to 0.97 ± 0.07 A/m^2^ and the power density increased tenfold to 710 ± 40 mW/m^2^. When the electron-carrier humic acid was added to base case #2 reactor at 0.5%, the current density increased 21-fold to 2.1 ± 0.4 A/m^2^ and the power density increased 21-fold to 1400 ± 200 mW/m^2^. Moreover, when the humic acid concentration was increased to 3.3% (i.e., the limit of solubility in water), the current density increased 60-fold to 6 ± 1 A/m^2^ (highest current density was 73-fold to 7.3 A/m^2^) and the power density increased 69-fold to 4700 ± 800 mW/m^2^ (highest power density was 77-fold to 5216 mW/m^2^) relative to base case #1 (Table 2). When the external resistance of 1 kΩ was removed, the current was measured as 750 µA yielding a current density of 15 A/m^2^ and power density was 10,688 mW/m^2^.

**Fig. 2:**
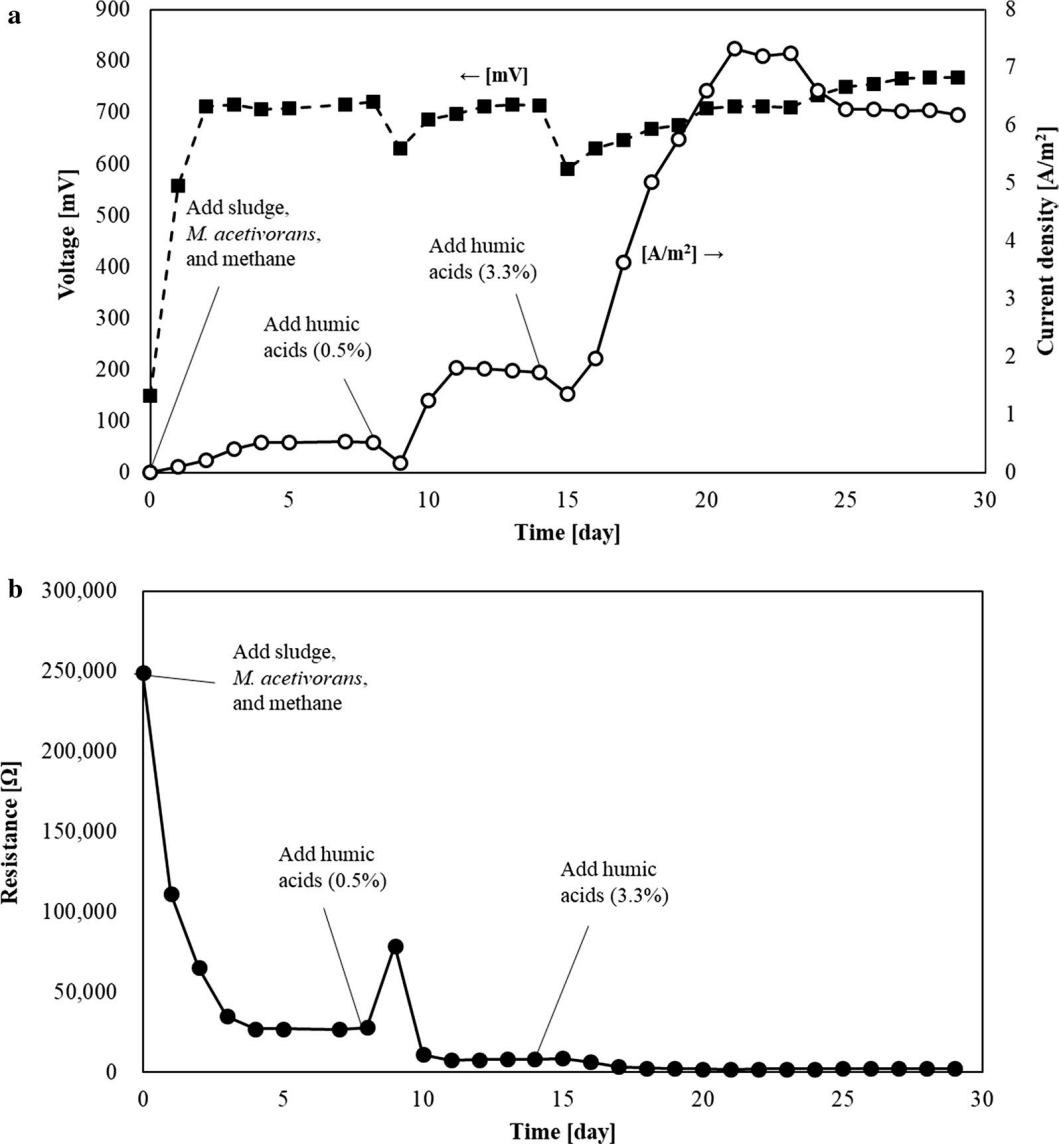
Voltage, current density, and resistance in the MFC. **a** Voltage (mV, black squares) and current density (A/m^2^, open circles) from one representative MFC (base case #2 + humic acids). The base case #2 consists of *G. sulfurreducens* added at the start and sludge, *M. acetivorans* AA*/*pES1MAT *mcr3* (“*M. acetivorans*”), and methane (100 mL/min, for 5 min) were added when the voltage became less than 150 mV. 0 on the abscissa indicates the time of adding sludge, *M. acetivorans* AA*/*pES1MAT *mcr3*, and methane. After 8 days, humic acids (conc. 0.5%) were added. After 14 days, the humic acid, concentration was increased to 3.3%. **b** Resistance from one representative MFC (base case #2 + humic acids) as calculated from the voltage and current (R = V/I) readings each day. Base case #2 consists of *G. sulfurreducens* added first to the anode followed by the addition of activated sludge, *M. acetivorans* AA*/*pES1MAT *mcr3* (“*M. acetivorans*”), and methane (100 mL/min, for 5 min) to the anode once the voltage was reduced to less than 150 mV (0 time point on the abscissa). After 8 days, humic acids (conc. 0.5%) were added, and the concentration was increased to 3.3% at day 14. The external resistance was 1000 Ohm

After 24 days, methane was refilled into the anode head space to investigate whether methane was limiting. However, current and voltage did not increase. These results show methane is not rate limiting. Critically, the resistance of the base case #2 + humic acids was dramatically decreased from 250,000 Ω (before adding the sludge and *M. acetivorans*) to 2000 Ω (after adding the humic acids at 3.3%) (Fig. 2b). These resistances include the external resistance (1000 Ω).

### Harnessing the elevated MFC power

To demonstrate the potential of the MFC with added electron carriers, we utilized three MFCs in series: base case #1 (10 mM acetate), base case #1 (0.5% humic acids), and base case #2 (3.3% humic acids) to increase the potential to 1700 mV and stored the electrons in a 10 F capacitor for 16 h. Using this stored energy, we were able to power a fan more than 1 min (Additional file 2: Video, see also http://www.che.psu.edu/faculty/wood/group/outreach/microbial-fuel-cell.html).

## Discussion

There are four points of operation that are possible to become rate limiting in our MFC (Fig. 1): (i) electron transfer by electron carriers to the anode, (ii) electron production from methane by *M. acetivorans* AA/pES1MAT *mcr3*, (iii) electron production from acetate by *Geobacter* spp., and (iv) electron transfer to the cathode. In this paper, we determined that the rate-limiting step was electron transfer to the anode by electron carriers because adding cytochrome C from *S. cerevisiae* as an electron carrier was beneficial for increasing the voltage by 3.4% (Table 1). More conclusively, by reducing the size of the cathode and by adding humic acids (3.3%, limit of solubility in water), the current density and power density was increased 60-fold to 6 ± 1 A/m^2^ and 69-fold to 4700 ± 800 mW/m^2^ compared to no humic acids and the larger cathode (Table 2). These values for current density and power density are based on the cathode surface area of 227 × 10^−5^ m^2^ (base case #1) or 5 × 10^−5^ m^2^ (base case #2). We do not normalize based on the brush anode size due to its large surface area that is difficult to calculate.

In contrast, changing the medium had little impact on the performance of the MFC; for example, adding sodium acetate for *G. sulfurreducens* was not beneficial because there was no increase in voltage (Table 1), and there was no benefit to the voltage of adding increased levels of trace elements, vitamins, cysteine–HCl, and Na_2_S (Table 1). In fact, adding additional cysteine–HCl and Na_2_S reduced the current density to 0.007 and 0.006 A/m^2^. Therefore, these components acted as a resistance. If the compounds were toxic, a voltage drop due to cell death would occur because adding sludge (bacteria) increases the voltage in our MFC by increasing the driving force for electricity [21]; because this voltage drop was not seen, we conclude that these components in excess inhibit electron transfer. Moreover, although the voltage without cysteine–HCl was higher than with cysteine–HCl, the current density in the absence of cysteine–HCl was lower (0.07 A/m^2^ vs. 0.1 A/m^2^ for the base case #1). Also, if cysteine–HCl was toxic, current should increase upon removing this medium component because removing toxic compounds increases current [22], but current density did not increase. Therefore, too high or too low amounts of cysteine–HCl are not advantageous for producing electricity.

From the viewpoint of resistance, adding more medium components increased the resistance (see Additional file 1: Table S1 for the medium composition). For example, adding additional cysteine–HCl and Na_2_S increased the resistance approximately 15- to 20-fold higher than base case #1, and adding additional sodium acetate increased the resistance twofold higher than base case #1. Also, removing cysteine–HCl increased the resistance twofold higher than base case #1 (Additional file 1: Table S2). Therefore, the large changes in medium composition here did not lead to increased electricity and had minimal influence on voltage; however, refined experiments may be warranted to explore this dependence more carefully. In contrast, adding a small dose of humic acids (0.5%) decreased the resistance of the base case #1 (Additional file 1: Table S2) and improves electricity generation.

Our maximum value of 7.3 A/m^2^ using methane as the substrate compares well with other MFCs that used more readily available (i.e., non-gaseous) substrates, including those utilizing *Geobacter* spp. For example, the maximum current density of *G. sulfurreducens* PCA using acetate and hydrogen as a substrate was 1.1 A/m^2^ [3] and 4.6 A/m^2^ using acetate [23]. In addition, *G. anodireducens* SD-1 using acetate as a substrate achieved 5.3 A/m^2^ [24], *G. soli* GSS01 using acetate as a substrate obtained 1.4 A/m^2^ [25], and *G. metallireducens* using acetate as substrates had 0.13 A/m^2^ [26].

The highest current density obtained in a MFC is 7.4 A/m^2^ using acetate as a substrate and *G. sulfurreducens* KN400 [27]. This system used an external resistance of 560 Ω. If we had used 560 Ω rather than 1000 Ω, we calculate that our methane MFC would be 9.5 A/m^2^ (Additional file 2); hence, by adding additional electron carriers we have created one of the best MFC systems. Also, power densities have been obtained as high as 7200 mW/m^2^ using glucose as a substrate and *Escherichia coli* K12 [11], 4310 mW/m^2^ using glucose as a substrate and an anaerobic bacterial consortium [28], and 3900 mW/m^2^ using acetate as a substrate and *G. sulfurreducens* KN400 [27]. The maximum power density of our MFC was 5216 mW/m^2^ (Additional file 1), which is 77-fold higher than the base case #1 (68 mW/m^2^). Also, if this system used an external resistance of 560 Ω, we calculate that our methane MFC would be 6769 mW/m^2^ (Additional file 1). Therefore, the power of our MFC is comparable to the best for any MFC.

The resistance of the base case #2 MFC was high (250,000 Ω) before adding sludge, which reduced the resistance to 27,000 Ω; hence, electron transfer from the sludge microorganisms to the electrode was substantially increased by adding sludge. In addition, by adding the electron carrier humic acids, the system resistance was decreased further to 2000 Ω (Fig. 2b) and current was increased (Fig. 2a). Therefore, electron carriers are limiting the MFC, and it should be possible to reduce the MFC system resistance by another 1000 Ω, so current density and power density may be increased further to approximately 16 A/m^2^ and 12,700 mW/m^2^ (with 1000 Ω external resistance) (Additional file 1). Furthermore, the structure of humic acids may be important for reducing the resistance since humic acids contain benzene and phenol groups [29], and these aromatic groups may support electron transfer by their *π* stacks or self-assembly [30].

Also, we calculated the Coulombic efficiency (CE) using our best-performing MFC system (base case #2 with 3.3% humic acids) as 82.3% over 78 days. This value was estimated using the following equation [8]:

$$ {\text{CE}}\, = \,\frac{{\int_{0}^{t} {I {\text{dt}}} }}{e  n  F}\, \times \,100, $$

where *I* is the MFC current we measured, t is time from after recharging with methane, *e* is the moles of electrons from each mole of methane consumed (i.e., each methane produces 8 electrons), *n* is the total methane consumed (mol), and F is the Faraday’s constant (96,485 s mA/mmol). This value corroborates our previous CE value of 90 ± 10% [8].

## Conclusions

In this study, we focused on improving electricity generation in a methane MFC. By varying reactor conditions including the medium, consortial members, electron carriers, cathode size, and inoculation order, we determined that electron carriers limit the current density and power generation and that the order of strain addition to the anode compartment is important. Specifically, electricity generation was improved 77-fold to 5216 mW/m^2^ by adding humic acids as an electron carrier in the MFC and by reducing the surface area of the cathode. Hence, we determined that in our system, the electron carrier was limiting electricity production rather than nano-wires. Hence, the methane MFC described here produces power at the highest level seen for all substrates utilized in any MFC.

## Methods

### Bacterial strains and growth conditions

The strains used in this study are listed in Table 3. *M. acetivorans* AA/pES1MAT *mcr3* was cultured routinely (not in the MFC) in HSYE medium (HS medium [31] with 2.5 g/L yeast extract) with 125 mM methanol as the carbon source and 2 µg/mL puromycin (to maintain plasmid pES1MAT *mcr3*) at 37 °C under anaerobic conditions (72% N_2_/18% CO_2_/10% H_2_). *G. sulfurreducens* PCA was grown routinely in anaerobic tubes on Geobacter basal medium [32] with 10 mM sodium acetate as the electron donor and 40 mM sodium fumarate as the electron acceptor. *G. metallireducens* GS-15 was grown routinely in anaerobic tubes on ferric citrate (13.7 g/L), sodium acetate (2.5 g/L), and a nutrient medium (NaHCO_3_, 2.5 g/L; NH_4_Cl, 1.5 g/L; NaH_2_PO_4_·H_2_O, 0.69 g/L; KCl, 0.1 g/L; 0.1% (w/v) Na_2_WO_4_·H_2_O solution, 0.25 mL; total 1× each of trace element and vitamin solutions) [26]. The sludge from the Office of Physical Plant at the Pennsylvania State University was cultured in HS medium including ferric ion and acclimated to methane under methane gas conditions at 37 °C [8]. *D. vulgaris* Hildenborough (ATCC 29579) was cultured routinely in modified Baar’s medium (ATCC 29579) with 0.025% sodium sulfide (as an oxygen scavenger); this culture was incubated at 30 °C without shaking [33].

**Table 3:** Strains used in this study

Strains	Description	Source
*M. acetivorans* AA/pES1MAT *mcr3*	Air-adapted *M. acetivorans* Amp^R^, Pur^R^, R6K *ori*, C2A *ori*, P_mcr_ANME-1_::*mcr*_ANME-1_	[12]
*G. sulfurreducens* PCA	Wild type	J. G. Ferry
*G. metallireducens* GS-15	Wild type	DSMZ 7210
*D. vulgaris* Hildenborough	Wild type	ATCC 29579

*Amp* ampicillin, *Pur* puromycin, *ANME-1* anaerobic methanotrophic archaeal population 1, *P*_*mcr_ANME-1*_ *mcr* promoter from ANME-1

### Microbial fuel cells

An H-type reactor was used for all MFC experiments [8]. One side bottle (155 mL volume) is for the anode and the other side bottle is for the cathode, and a Nafion 117 proton exchange membrane (Dupont, Wilmington, DE, USA) was clamped between these two bottles. To prevent leaking, a rubber gasket (35 mm outer diameter) was used with silicon grease (Dow, Midland, MI, USA). Before using the Nafion membrane, it was cut into 4 × 4-cm squares, placed in 30% hydrogen peroxide at 80 °C for 1 h, placed in distilled water at 80 °C for 1 h, placed in 0.5 M sulfuric acid at 80 °C for 1 h, and placed in distilled water at 80 °C for 1 h. The cathode was carbon cloth (Fuel Cell Store, Boulder, CO, USA); the full size was 38 mm in diameter (surface area is 0.00227 m^2^), and the small size was 5 mm × 5 mm (surface area is 50 × 10^−6^ m^2^). Using insulating tape [1 Mil Kapton Tape (Polyimide)—1/2” × 36 Yds, Dupont Wilmington, DE, USA], the carbon cloth was attached to a 10-cm-long titanium wire (1.0 mm diameter, Alfa Aesar, Haverhill, MA, USA). The anode was a brush electrode (Mill-Rose, Mentor, OH, USA) with carbon fibers (PANEX 35 50 K, Zoltek, St Louis, MO, USA) and was twisted onto two titanium wires [34] 12.7 cm long and heat treated at 450 °C for 30 min [35]. Platinum wire (0.25 mm diameter, 99.9%, #45093-BU, Alfa Aesar, Tewksbury, MA, USA) was cut to 2 cm lengths and attached to a titanium wire using insulating tape (active surface area 1 × 10^−5^ m^2^). These electrodes were inserted into the rubber septum (42 mm diameter) and placed into each MFC bottle using a plastic cap with a hole. Before setting the anode into the bottle, grease was applied to the top of anode bottle to completely seal it.

Cultures (200 mL) of *M. acetivorans* AA/pES1MAT *mcr3* and *G. sulfurreducens* were collected by centrifugation (at 3800*g* for 20 min); the pellets were washed three times using HS medium lacking resazurin (HSNR, Additional file 1: Table S1), resuspended in 100 mL of HSNR containing 2 µg/mL puromycin, and placed in the anode bottle. The cathode electrolyte solution was 100 mL of 100 mM potassium ferricyanide in 100 mM phosphate buffer containing 5.8 mM ammonium chloride and 1.7 mM potassium chloride (pH 7.0). Both anode and cathode caps were closed tightly. Methane (99.999% purity, catalog no. ME5.0RS, Praxair) was added into the anode chamber at 100 mL/min for 5 min. The MFC reactor was incubated at 30 °C, and the voltage was measured using a 16-channel differential analogue input module (NI 9205, National Instruments, Austin, TX, USA). Current was measured through the 1000 Ω external resistance using a commercial electric multimeter MAS830B (Home Depot, Atlanta, GA, USA). After the voltage was stable, i.e., when the potential between the anode and the cathode reached equilibrium (150 mV typically after 16–79 days), sludge was added. Sludge (4.5 mL) was centrifuged at 9600*g* for 1 min, and the pellet was resuspended in 2 mL of HSNR with 2 µg/mL puromycin. The re-suspended sludge was added to the anode using a syringe. Methane was re-charged into the anode headspace at 100 mL/min for 5 min upon sludge addition. The resistance (Ω) was calculated from Ohm’s law (R = V/I), and the current density (A/m^2^) was calculated using the cathode surface area.

### Additional components for the MFC

Two types of cytochrome C, from equine heart (#250600, EMD Millipore Fisher, Burlington, MA, USA) and from *S. cerevisiae* (#C2436, Sigma-Aldrich, St. Louis, MO, USA), were employed as electron carriers for the MFC. Each (25 mg) was dissolved in sterilized distilled water (3 mL), and the cytochrome C stock solution was added to the anode of MFC (final conc. 20 µM) with sludge or added after the MFC reached its maximum voltage. Humic acid sodium salt (#H16752, Sigma-Aldrich, St. Louis, MO, USA) was dissolved in HSNR (4 mL) with 2 µg/mL puromycin to make the stock solution and added into the anode of the MFC (final conc. 0.5 or 3.3%) with sludge or after the MFC reached its maximum voltage. Sodium acetate anhydrous (#071380, Fisher Scientific, Fair Lawn, NJ, USA) stock was prepared (1 M) and added to the anode of the MFC (final conc. 10 mM) with sludge. l-Cysteine hydrochloride (#C1276, Sigma-Aldrich, St. Louis, MO, USA) stock was prepared by dissolving 0.2 g in 0.2 mL sterilized distilled water and added to the anode of the MFC (final conc. 16 mM) after it reached the maximum voltage. Sodium sulfide nonahydrate (#S25570A, Fisher Science Education, Nazareth, PA, USA) stock was prepared at 100 mM, and 5 mL was added into anode of MFC (final conc. 5 mM) after it reached the maximum voltage. These stock solutions were made anaerobic before use by placing in an anaerobic chamber for at least 16 h.

### Powering a fan

Three MFCs and a 10-F capacitor (Catalog # BCAP0010 P270 T01, Maxwell Technologies Inc) were connected in series to charge the capacitor. Before charging the capacitor overnight, the capacitor was confirmed as empty using a multimeter. Using the full capacitor, a fan (Catalog # 7306, Hydrogen and Fuel Cell, Claremont, CA) was powered for over 1 min.

## Additional files

**Additional file 1.** Calculation for a 560 Ω external resistance or a 0 Ω internal resistance. Electric energy available in the methane MFC. **Table S1.** Composition of HSNR medium. **Table S2**. Resistance (Ω) for base case #1 with additional components.

**Additional file 2: Video.** Microbial fuel cell that reverses methanogenesis is used to convert methane into electricity to drive a fan.
