# Supplementary material for: Electron carriers increase electricity production in methane microbial fuel cells that reverse methanogenesis
Source: Biotechnol Biofuels. 2018 Jul 25;11:211. doi: 10.1186/s13068-018-1208-7 (PMC6058355; doi:10.1186/s13068-018-1208-7)
Supplement: Supplementary file 1 — Additional file 1. Calculation for a 560 Ω external resistance or a 0 Ω internal resistance. Electric energy available in the methane MFC. Table S1. Composition of HSNR medium. Table S2. Resistance (Ω) for base case #1 with additional components. [file 13068_2018_1208_MOESM1_ESM.docx]

**SUPORTING INFORMATION**

**Electron Carriers Increase Electricity Production in Methane Microbial Fuel Cells That Reverse Methanogenesis**

**Ryota Yamasaki^1^, Toshinari Maeda^2^ and Thomas K. Wood^1*^**

^1^Department of Chemical Engineering, Pennsylvania State University,

University Park, Pennsylvania, 16802-4400, USA

^2^Department of Biological Functions Engineering, Kyushu Institute of Technology,

2-4 Hibikino, Wakamatsu, Kitakyushu 808-0196, Japan

*For correspondence. E-mail [twood@engr.psu.edu](mailto:twood@engr.psu.edu)

Tel.(+)1 814-863-4811; Fax (1) 814-865-7846

**Running title:** Electrical carriers increase electricity in methane MFCs

**Keywords:** microbial fuel cells, anaerobic, *Geobacter*, methane, current density

**Calculation for a 560 Ω external resistance or a 0 Ω internal resistance.** The highest voltage was obtained in the base case #2 + humic acids MFC which had a maximum voltage of 712.5 mV and maximum current of 366 µA (**Fig. 2A**). For this case, R (Ω) = 712.5 mV/366 × 10^-3^ mA = 1,947 Ω, which includes the 1000 Ω external resistance. Therefore, the internal resistance is 947 Ω. The maximum current density in a MFC was obtained with a 560 Ω external resistance [[27](#_ENREF_27)]. For comparison, if the external resistance of our MFC was 560 Ω, our system resistance would be: R (Ω) = 1,947 – 1000 + 560 = 1,507 Ω. Because voltage that the anode produces does not change, I (µA) = 712.5 mV/1,507 Ω = 473 µA. Because our cathode surface area of cathode is 50 × 10^-6^ m^2^, the current density with 560 Ω resistance would be 473 µA/50 × 10^-6^ m^2^ = 9.5 A/m^2^. Also, the power density would be 712.5 mV × 9.5 A/m^2^ = 6,769 mW/m^2^. If the internal resistance approaches 0 Ω (i.e., there is only 1000 Ω external resistance), the current would be 795.9 µA (= 795.9 × 10^3^ µV/1000 Ω). Because our cathode surface area is 50 × 10^-6^ m^2^, the current density would be 795.9 µA/50 × 10^-6^ m^2^ = 15.9 A/m^2^. Also, the power density would be 0.8 V × 15.9 A/m^2^ = 12.7 W/m^2^.

**Electric energy available in the methane MFC.** A 155 mL bottle is used for the anode, and it is filled with 100 mL of culture; hence, there are 55 mL of headspace filled with methane, which is 1.5 × 10^21^ molecules [1 mol of gas is 22.4 L at standard conditions, so 0.055 L/(22.4 L/mol) x 6.022 × 10^23^ molecules/mol].

For the anode reaction, 4CH_4_ + 2HCO_3_^−^ → 3CH_3_COO^−^ + 8e^−^ + 9H^+^; hence, 1 mole of methane can produce 2 mole of electrons. So, if all the methane reacts, 3.0 × 10^21^ electrons are produced.

In our MFC, the maximum current is approximately 366 µA. 366 µA = 366 µC/s. 1 electron = 1.6 × 10^-19^ C. So, (366 µC/s)/(1.6 × 10^-19^ C) = 2.3 × 10^15^ electrons react/s at the cathode. Hence, the amount of time the MFC can provide power is (3.0 × 10^21^ electrons)/(2.3 × 10^15^ electrons/s) = 1.3 × 10^6^ s or 15 days.

Our best MFC produced 713 mV and 366 × 10^-6^ A. Hence, the electric power may be calculated as P = V × I = 713 × 10^-3^ V × 366 × 10^-6^ A = 256 µW. The power density may be calculated as P divided by surface area of cathode electrode (50 × 10^-6^ m^2^) = 261 × 10^-6^ W/50 × 10^-6^ m^2^ = 5,216 mW/m^2^. The total energy may be calculated as W = V × I × t = 713 × 10^-3^ V × 366 × 10^-6^ A × 1.3 × 10^6^ s = 339 W·s (or 339 J). Hence, our MFC can produce 256 µW of power for 15 days and contains a total of 339 J of energy.

**Table S1.** Composition of HSNR medium.

| **Chemical** | **Final concentration (mM)** | **Chemical** | **Final concentration (mM)** |
| --- | --- | --- | --- |
| NaCl | 400 | Puromycin dihydrochloride | 0.0037 |
| MgCl_2_.6H_2_O | 54 | ^a^CoSO_4_.7H_2_O | 0.0036 |
| NaHCO_3_ | 45 | ^a^FeSO_4_.7H_2_O | 0.0036 |
| NH_4_Cl | 19 | ^b^Lipoic acid | 0.0024 |
| KCl | 13 | ^a^Boric acid | 0.0016 |
| KH_2_PO_4_ | 5.0 | ^b^Thiamine hydrochloride | 0.0015 |
| Cysteine.HCl | 3.2 | ^b^Riboflavin | 0.0013 |
| CaCl_2_.2H_2_O | 2.0 | ^b^Pantothenic acid (Ca salt) | 0.0010 |
| Na_2_S.9H_2_O | 1.0 | ^a^Na_2_MoO_4_.2H_2_O | 0.0010 |
| ^a^MgSO_4_.7H_2_O | 0.10 | ^a^NiCl_2_.6H_2_O | 0.0010 |
| ^a^Nitrilotriacetic acid | 0.079 | ^b^Biotin | 0.00082 |
| ^a^MnSO_4_.H_2_O | 0.030 | ^b^Folic acid | 0.00045 |
| ^a^ZnCl_2_ | 0.0059 | ^a^CuSO_4_.5H_2_O | 0.00040 |
| ^b^Pyridoxine hydrochloride | 0.0049 | ^a^KAl(SO_4_)_2_.12H_2_O | 0.00021 |
| ^b^Nicotinic acid | 0.0041 | ^b^Vitamin B_12_.HCl | 0.0000074 |
| ^b^*p*-Aminobenzoic acid | 0.0037 |  |  |

^a^Trace element solution, ^b^Vitamin solution

**Table S2.** Resistance (Ω) for base case #1 with additional components. These resistances occurred during the time of maximum voltage and current.

| **Base case #1** | **Base case #1 + cysteine-HCl (16 mM)** | **Base case #1 + Na_2_S (5 mM)** | **Base case #1 + Sodium acetate (10 mM)** | **Base case #1 + Humic acids (0.5 %)** | **Base case #1 without cysteine-HCl** |
| --- | --- | --- | --- | --- | --- |
| 2,398 | 35,168 | 46,719 | 4,803 | 2,225 | 4,885 |
